# Quality assurance in severe sepsis: an individualised audit/feedback system results in substantial improvements in sepsis care at a large UK teaching hospital

**DOI:** 10.1186/cc12960

**Published:** 2013-11-05

**Authors:** Mark Simmonds, Esme Blyth, Marc Chikhani, Jaimie Coleman, Vivienne Weston, Tim Hills

**Affiliations:** 1 Sepsis Action Group, Nottingham University Hospitals NHS Trust, Nottingham, UK

## Background

Severe sepsis has a high mortality and high healthcare costs. Rapid recognition and treatment can save lives but requires a coordinated response [1]. Hospital-wide audits in 2005 and 2010 showed significant deficiencies when compared with international guidelines, with 35% of cases receiving antibiotics in <1 hour and only 25% receiving basic pre-ICU interventions in a timely manner. By time-lining our response to severe sepsis, we identified system and process failures [2]. Some system improvements (for example, providing first-line antibiotics in acute areas) were straightforward to tackle, but sepsis care remained reliant on individual clinician response. Equally, whilst dissemination of organisation-level audit data raised the profile of sepsis, it appeared that individual clinicians did not view it as 'their problem'. It is recognised that individualised feedback can improve care, as pride and the competitive nature of healthcare workers drives improvement. This is especially true when adherence to recommended practice is low [3]. We tried to change behaviour by creating a rapid response audit/feedback mechanism that informed clinicians of their own response to the severely septic patient, from which they could learn and improve.

## Materials and methods

**Figure 1:**
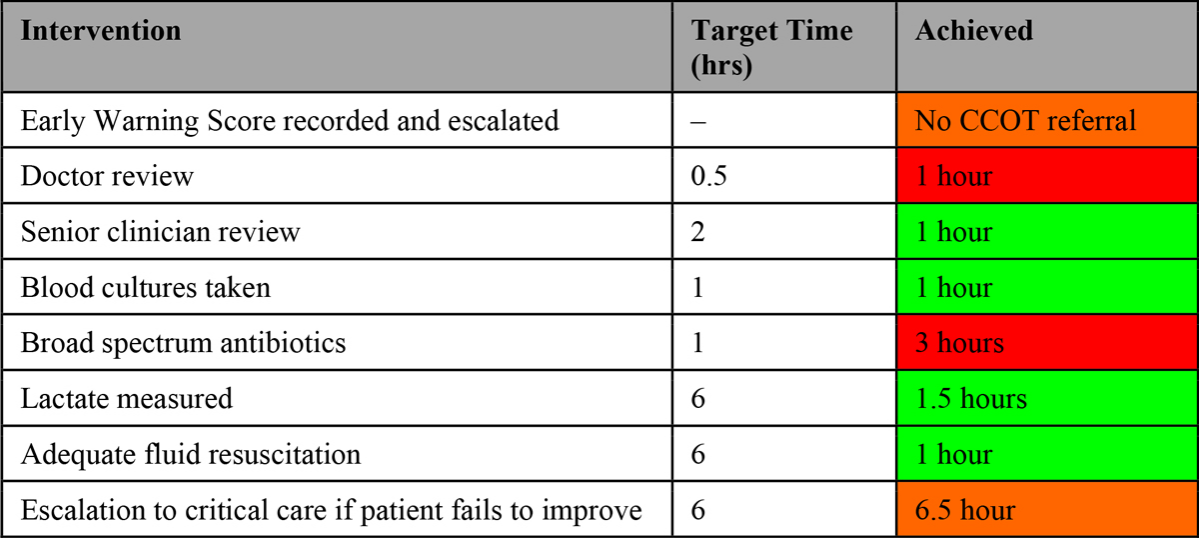
**Example report**.

Patients admitted to any critical care unit (58 beds, four units, two sites) with a primary admission diagnosis of infection were screened for severe sepsis. The pre-ICU care of patients who met the criteria was then audited against the Surviving Sepsis Guidelines [1]. Time zero was defined as when the criteria for severe sepsis were first met. Information on timings of key interventions (such as doctor review and request for critical care escalation) was also gathered. An individualised traffic-light report was then generated and emailed to the patient's consultant and other stakeholders such as junior doctors or nurses involved in the patient's care (Figure 1). We aimed to report cases back within 7 days of arrival to ensure the patient story was fresh in the clinician's mind. A cumulative report is generated monthly to track organisation-wide performance.

## Results

**Figure 2:**
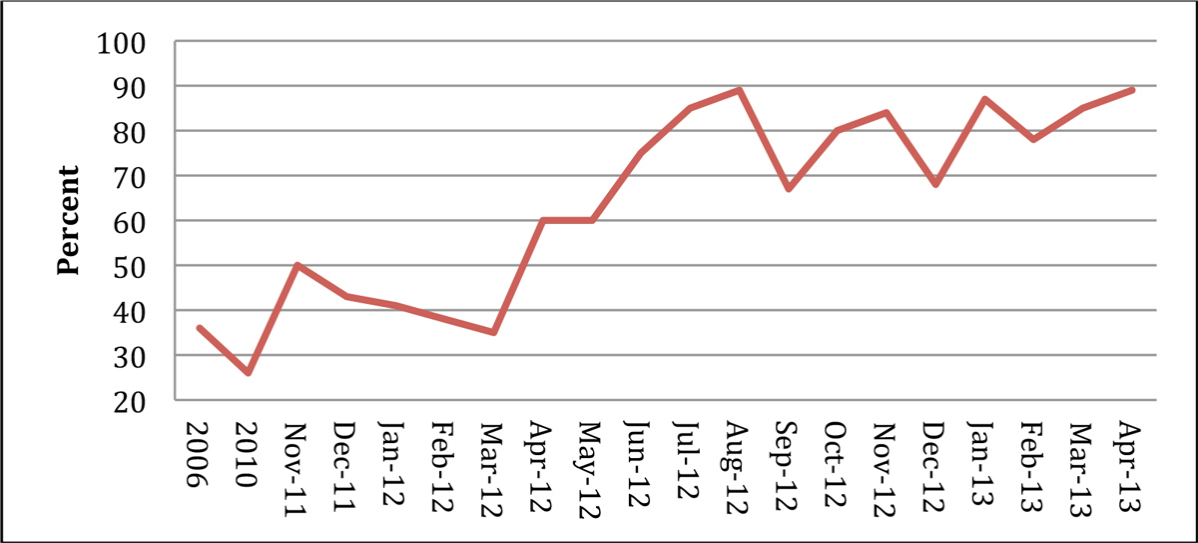
**Compliance with antibiotics in <1 hour**.

**Figure 3:**
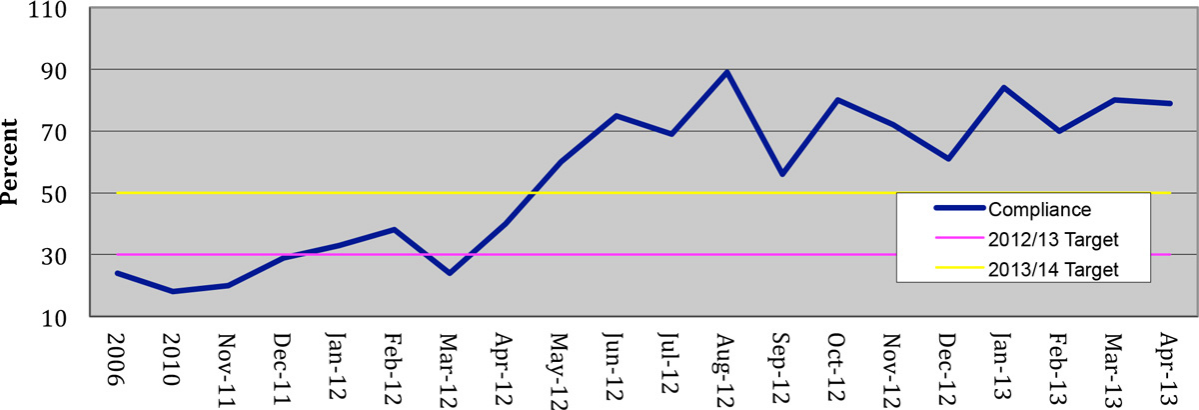
**Compliance with pre-ICU bundle of care**.

Since November 2011 we have provided feedback on over 300 severe sepsis cases. Antibiotic administration in <1 hour has risen from 35% to 75% (Figure 2), and pre-ICU bundle compliance has risen from 25% to 70% (Figure 3). Since November 2012 all sepsis cases in our critical care units have been audited (30 to 35 cases/month).

## Conclusions

Individualised feedback on sepsis care has led to substantial improvements in guideline compliance.
